# Temperature-dependent Spike-ACE2 interaction of Omicron subvariants is associated with viral transmission

**DOI:** 10.1128/mbio.00907-24

**Published:** 2024-07-02

**Authors:** Mehdi Benlarbi, Shilei Ding, Étienne Bélanger, Alexandra Tauzin, Raphaël Poujol, Halima Medjahed, Omar El Ferri, Yuxia Bo, Catherine Bourassa, Julie Hussin, Judith Fafard, Marzena Pazgier, Inès Levade, Cameron Abrams, Marceline Côté, Andrés Finzi

**Affiliations:** 1 Centre de Recherche du CHUM, Montréal, Québec, Canada; 2 Département de Microbiologie, Infectiologie et Immunologie, Université de Montréal, Montréal, Québec, Canada; 3 Montreal Heart Institute, Research Center, Montreal, Quebec, Canada; 4 Department of Biochemistry, Microbiology and Immunology, Centre for Infection, Immunity and Inflammation, University of Ottawa, Ottawa, Ontario, Canada; 5 Département de Médecine, Université de Montréal, Montréal, Québec, Canada; 6 Mila—Quebec AI institute, Montreal, Quebec, Canada; 7 Laboratoire de Santé Publique du Québec, Institut National de Santé Publique du Québec, Sainte-Anne-de-Bellevue, Québec, Canada; 8 Infectious Disease Division, Department of Medicine of Uniformed Services University of the Health Sciences, Bethesda, Maryland, USA; 9 Department of Biochemistry and Molecular Biology, Drexel University College of Medicine, Philadelphia, Pennsylvania, USA; McMaster University, Hamilton, Ontario, Canada

**Keywords:** SARS-CoV-2, Omicron subvariants, humoral responses, ACE2 binding, temperature

## Abstract

The continued evolution of severe acute respiratory syndrome 2 (SARS-CoV-2) requires persistent monitoring of its subvariants. Omicron subvariants are responsible for the vast majority of SARS-CoV-2 infections worldwide, with XBB and BA.2.86 sublineages representing more than 90% of circulating strains as of January 2024. To better understand parameters involved in viral transmission, we characterized the functional properties of Spike glycoproteins from BA.2.75, CH.1.1, DV.7.1, BA.4/5, BQ.1.1, XBB, XBB.1, XBB.1.16, XBB.1.5, FD.1.1, EG.5.1, HK.3, BA.2.86 and JN.1. We tested their capacity to evade plasma-mediated recognition and neutralization, binding to angiotensin-converting enzyme 2 (ACE2), their susceptibility to cold inactivation, Spike processing, as well as the impact of temperature on Spike-ACE2 interaction. We found that compared to the early wild-type (D614G) strain, most Omicron subvariants' Spike glycoproteins evolved to escape recognition and neutralization by plasma from individuals who received a fifth dose of bivalent (BA.1 or BA.4/5) mRNA vaccine and improve ACE2 binding, particularly at low temperatures. Moreover, BA.2.86 had the best affinity for ACE2 at all temperatures tested. We found that Omicron subvariants’ Spike processing is associated with their susceptibility to cold inactivation. Intriguingly, we found that Spike-ACE2 binding at low temperature was significantly associated with growth rates of Omicron subvariants in humans. Overall, we report that Spikes from newly emerged Omicron subvariants are relatively more stable and resistant to plasma-mediated neutralization, present improved affinity for ACE2 which is associated, particularly at low temperatures, with their growth rates.

**IMPORTANCE:**

The persistent evolution of SARS-CoV-2 gave rise to a wide range of variants harboring new mutations in their Spike glycoproteins. Several factors have been associated with viral transmission and fitness such as plasma-neutralization escape and ACE2 interaction. To better understand whether additional factors could be of importance in SARS-CoV-2 variants’ transmission, we characterize the functional properties of Spike glycoproteins from several Omicron subvariants. We found that the Spike glycoprotein of Omicron subvariants presents an improved escape from plasma-mediated recognition and neutralization, Spike processing, and ACE2 binding which was further improved at low temperature. Intriguingly, Spike-ACE2 interaction at low temperature is strongly associated with viral growth rate, as such, low temperatures could represent another parameter affecting viral transmission.

## INTRODUCTION

**Fig 1:**
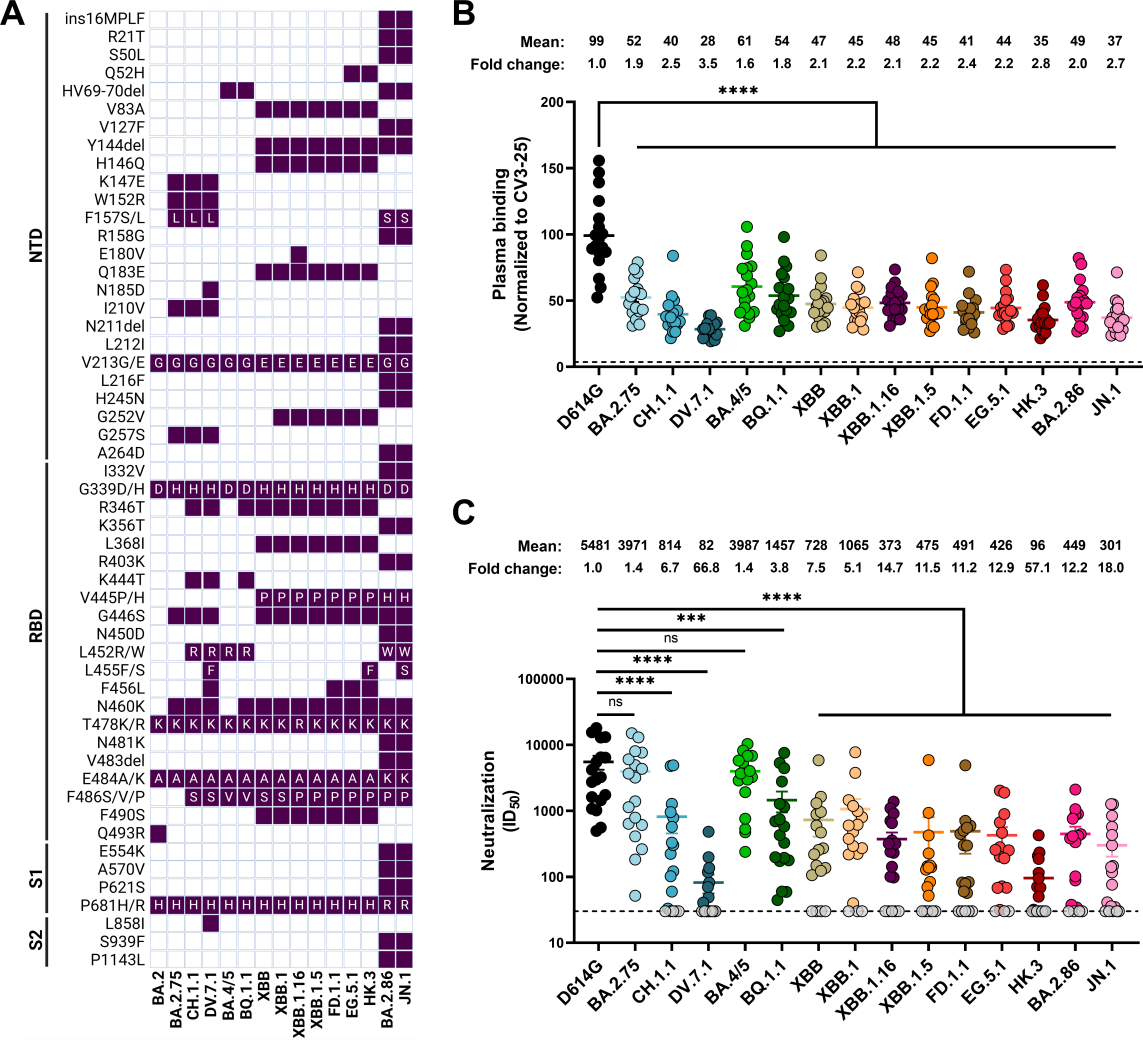
Plasma recognition and neutralization of emerging Omicron subvariants. (**A**) Defining Spike glycoprotein (S) mutations of recent Omicron subvariants in the N-terminal domain (NTD), receptor-binding domain (RBD), and the rest of subunit 1 (S1) and subunit 2 (S2). Omicron subvariants’ mutations are in reference to BA.2, only mutations differing from BA.2 are shown. (**B**) HEK293T cells were transfected with the indicated full-length S, stained with the CV3-25 mAb or with plasma from vaccinated individuals collected 3-4 weeks following a fifth dose of mRNA vaccine, and analyzed by flow cytometry. The values represent the MFI normalized by CV3-25 mAb binding. (**C**) Neutralization activity was measured by incubating pseudoviruses bearing SARS-CoV-2 Spike glycoproteins with serial dilutions of plasma from vaccinated individuals. Neutralization half-maximal inhibitory serum dilution (ID_50_) values were determined using a normalized nonlinear regression using GraphPad Prism software. Limits of detection are plotted as a dashed line (***P < 0.001; ****P < 0.0001; ns, non-significant).

**Fig 2:**
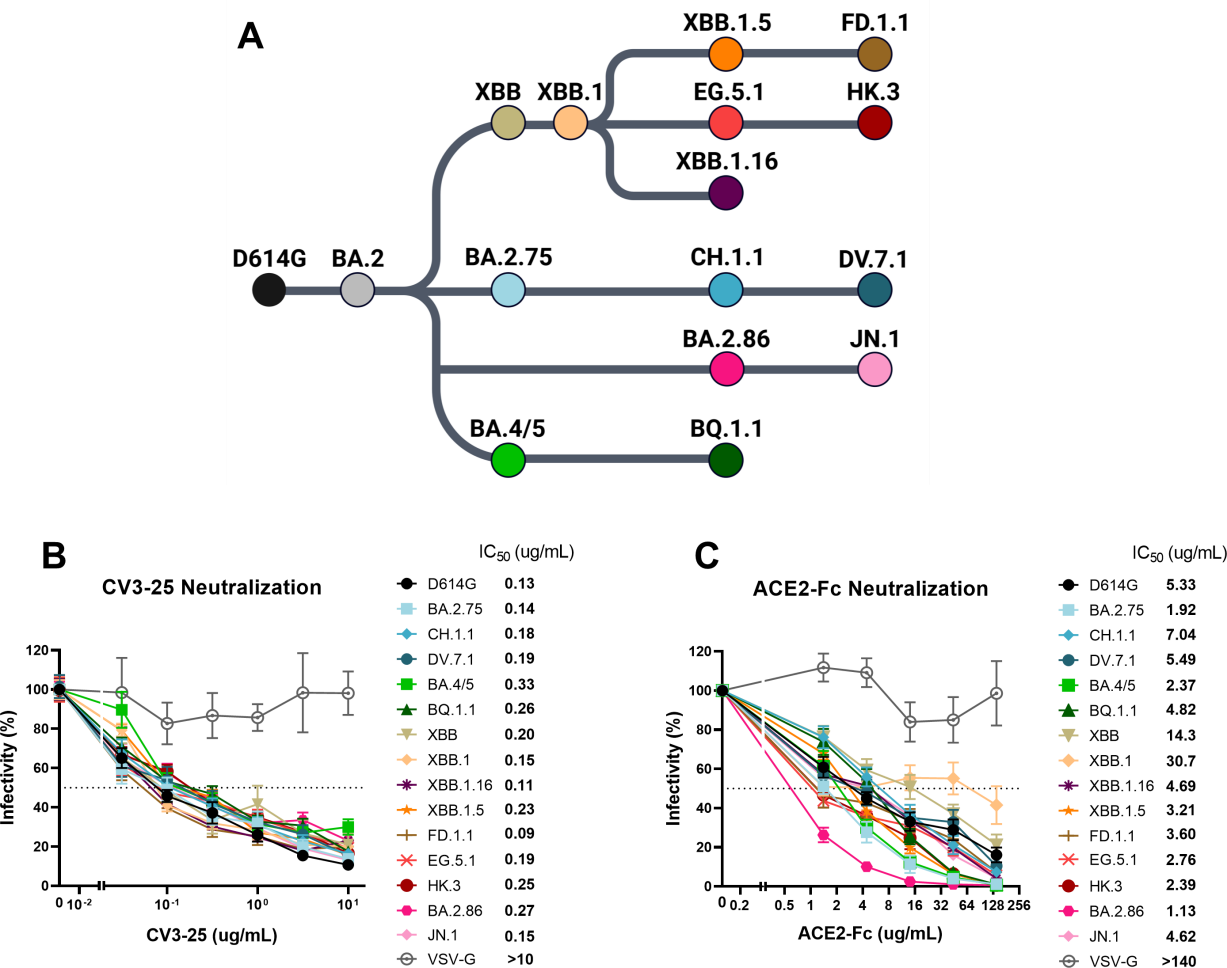
Evolution of Omicron subvariants and their susceptibility to neutralization by CV3-25 mAb and ACE2-Fc. (**A**) Schematics showing the evolving Omicron sublineages of predominant strains. (**B and C**) Neutralization activity was measured by incubating pseudoviruses bearing SARS-CoV-2 Spike glycoproteins with serial dilutions of anti-S2 CV3-25 monoclonal antibody (**B**) or ACE2-Fc (**C**). Neutralization half-maximal inhibitory concentration (IC_50_) values were determined using a normalized nonlinear regression using GraphPad Prism software.

Since the beginning of the coronavirus disease 2019 (COVID-19) pandemic, multiple severe acute respiratory syndrome 2 (SARS-CoV-2) variants emerged, raising concerns about the effectiveness of infection and/or vaccine-elicited immunity (1–5). The emergence of a SARS-CoV-2 variant harboring 33 mutations in its Spike glycoprotein (S), Omicron (BA.1), reduced vaccine efficacy against infection due to its improved antibody escape (6–12). While modified versions of mRNA vaccines were produced to induce an immune response against the Omicron Spike (BA.1) (13, 14) and its subvariants (15–18), the persistent evolution of SARS-CoV-2 gave rise to various subvariants across the world (Fig. 1A and 2A) (19). BA.1 was rapidly surpassed by BA.2 (20), and since then, several of its progenies have emerged and demonstrated improved transmission (21). Notably, BA.2.75, which surfaced in May 2022, further mutated into CH.1.1 (22, 23). On the other hand, BA.4 and BA.5, which harbor the same Spike, further evolved to BQ.1.1 in late 2022, showing improved immune escape ability (14, 16, 24). In that same period, a recombinant sublineage, XBB, emerged and showed enhanced immune escape (25, 26). Since then, most newly occurring Omicron subvariants are derived from XBB and carry the Ser486Pro mutation known to enhance the affinity for the human receptor angiotensin converting enzyme 2 (ACE2) (27), such as XBB.1.5 which quickly dominated over XBB in January 2023 (28, 29). Recently, the main XBB subvariants were XBB.1.5, XBB.1.9.1, XBB.1.16, XBB.2.3, and EG.5.1, representing around 80% of reported viral sequences (30) in addition to EG.5.1 sublineages HK.3 and HV.1, which are growing rapidly around the globe (31, 32). In August 2023, the emergence of BA.2.86, a highly divergent BA.2 subvariant, caused great concerns regarding infection- and vaccine-elicited immune responses (30, 33, 34). Although the global number of infections related to BA.2.86 were relatively limited, its fast expansion and diversification in various countries has been noted, with the emergence of BA.2.86 sublineages (i.e., JN.1, JN.2, and JN.3) showing enhanced transmission globally (32, 35). As of January 2024, JN.1 and its derivatives represent around 70% of reported viral sequences according to Nextstrain (https://nextstrain.org/ncov/gisaid/global/6m).

The Spike glycoprotein is a metastable fusion protein composed of a trimer of heterodimers expressed at the surface of viral particles and can also be detected at the surface of infected cells (36–39). Its interaction with the receptor ACE2 on host cells enables S cleavage by host proteases, thus exposing the fusion peptide leading to viral entry (40–45). Given that the Spike glycoprotein is one of the main targets of humoral responses elicited by SARS-CoV-2 infection and vaccines, a strong selective immune pressure against this crucial protein led to the current evolution of emerging Omicron subvariants (21, 46–49). Each of these subvariants has acquired mutations in Spike that help evade humoral responses, resulting, in some cases, in increased binding affinity for ACE2 (50–52). Interestingly, multiple Omicron lineages gained identical or similar Spike mutations in key antigenic sites in the receptor binding domain (RBD) and in the N-terminal domain (NTD), suggesting a convergent evolution (53, 54). It is now well established that Spike evolution is intimately associated with viral fitness and transmission in humans (5, 50).

While viral transmission is a multifactorial phenomenon, Spike-ACE2 interaction appears to play an predominant role (19, 40, 50). As such, parameters influencing the strength of this interaction may influence viral growth of emerging Omicron subvariant. We previously demonstrated that temperature modulates the interaction between SARS-CoV-2 Spike and ACE2, with temperatures lower than 37°C (i.e., 4°C and 25°C) increasing ACE2-binding affinity and viral entry (55). We showed that this modulation was explained by favorable thermodynamic changes leading to the stabilization of the RBD-ACE2 interface and by triggering a more “open’’ conformation of the Spike trimer. Subsequent work on early Omicron subvariants (BA.1, BA.2, BA.2.12.1, BA.4/5) also showed an impact of temperature on Spike-ACE2 interaction (56). This is of particular interest because it has been suggested that the optimal air temperature for SARS-CoV-2 transmission ranges from 5°C to 15°C (57, 58). Furthermore, SARS-CoV-2 airway transmission is influenced by external factors, such as the temperature gradient that exists in human airways (from 30°C to around 36°C) (59, 60). Hence, external temperatures reaching sub-zero degrees Celsius might prime SARS-CoV-2 Spike for ACE2 binding, thus improving its adsorption onto the epithelial airway cells (55, 61). Interestingly, several studies have observed a link between colder temperatures and a higher incidence of COVID-19 cases (62, 63), and this could also be another factor underlying SARS-CoV-2 seasonality (64, 65).

To better understand parameters associated with viral transmission, we functionally characterized the Spike glycoprotein of emerging Omicron subvariants, which possess enhanced immune escape and increased binding affinity to ACE2 compared to early Omicron strains. We tested the capacity of plasma from individuals who received a fifth dose of bivalent (BA.1 or BA.4/5) mRNA vaccine to recognize and neutralize several Spikes from recent Omicron subvariants. We next determined how temperature affects the interaction between Spike and ACE2 by combining an array of biochemical and biological assays, including biolayer interferometry, flow cytometry, and virus capture assay. We also evaluated the susceptibility of Omicron subvariants to cold inactivation, a property that may reflect the propensity of Spikes to ensue conformational transitions (66–68). The associations between these parameters and the viral growth rate of each Omicron subvariant in the population between early 2022 and early 2024 were evaluated.

## MATERIALS AND METHODS

### Human participants

The study was conducted with samples from 18 individuals, 11 females, and 7 males (age range: 51–64 years). Blood samples were collected 22 days after the fifth dose of mRNA vaccine. No specific criteria such as sex, clinical, or demographic were used for inclusion to the cohort. Out of the 18 participants, 9 had detectable levels of anti-nucleocapsid antibody levels, suggesting a recent breakthrough infection. All participants received either the Moderna or Pfizer mRNA monovalent (WT) vaccines prior to the fifth dose. Characteristics of the cohort are summarized in Table 1.

**TABLE 1:** Characteristics of the SARS-CoV-2 vaccinated cohort

	Vaccinated cohort
Number (*n*)^*a*^	18
Age^*b*^	59 (51–64)
Sex^*a*^	
Female (*n*)	11
Male (*n*)	7
Days between the 4th and 5th doses^*b*^	148 (129–156)
Recent breakthrough infection (*n*)	9
Fifth dose (*n*)^*a*^	
Pfizer monovalent	2
Moderna monovalent	0
Pfizer BA.4/5	13
Moderna BA.1	3
Days between the fifth dose and sample collection^*b*^	22 (21–27)

^*a*^ Values displayed are numbers.

^*b*^ Values displayed are medians, with interquartile ranges in parentheses.

### Plasmids

The plasmids expressing SARS-CoV-2 Spike D614G, and SARS-CoV-2 RBD_WT_ (residues 319–541) fused with a 6xHis-tag were previously described (69). The RBD sequence (encoding for residues 319–541) fused to a C-terminal 6xHis-tag was cloned into the pcDNA3.1(+) expression vector. The plasmids encoding the full-length Spike from BA.2.75, CH.1.1, DV.7.1, BA.4/5, BQ.1.1, XBB, XBB.1, XBB.1.16, XBB.1.5, FD.1.1, EG.5.1, HK.3, BA.2.86, and JN.1 variants were generated by overlapping PCR using a codon-optimized wild-type SARS-CoV-2 Spike gene that was synthesized (Biobasic, Markham, ON, Canada) and cloned in pCAGGS as a template (8, 16, 56, 70, 71). All constructs were validated by Sanger sequencing. The plasmid encoding for soluble human ACE2 (residues 1–615) fused with an 8xHis-tag was reported elsewhere (37). The plasmid encoding for the ACE2-Fc chimeric protein, a protein composed of an ACE2 ectodomain (1–615) linked to an Fc segment of human IgG1, was previously reported (72). The lentiviral vector pNL4.3 R^-^E^−^ Luc was obtained from NIH AIDS Reagent Program. The vesicular stomatitis virus G (VSV-G)-encoding plasmid was previously described (73). The plasmid coding for the anti-S2 CV3-25 mAb was previously described (74) and its structure and sequences are available on protein databank (PDB: 7RAQ_L, PDB: 7RAQ_H) and on GenBank (GenBank: MW681575.1, GenBank: MW681603.1).

### Cell lines

HEK293T cells (obtained from American Type Culture Collection [ATCC]) were derived from 293 cells, into which the simian virus 40 T-antigen was inserted. HEK293T-ACE2 were previously described (46) and were maintained in the presence of puromycin (2 µg/mL). Cf2Th cells (ATCC) are canine thymocytes resistant to SARS-CoV-2 entry and were used as target cells in the virus capture assay. HEK293T cells and Cf2Th were maintained at 37°C under 5% CO2 in Dulbecco’s modified Eagle’s medium (DMEM) (Wisent, Saint-Jean Baptiste, QC, Canada), supplemented with 5% fetal bovine serum (FBS) (VWR, Radnor, PA, USA) and 100 µg/mL penicillin/ streptomycin (Wisent).

### Protein expression and purification

Proteins and CV3-25 were expressed and purified as described (55). A detailed description is provided in the supplemental material.

### Flow cytometry analysis of cell-surface staining

Cell-surface staining of Spike glycoproteins was assessed by flow cytometry, as described (55). A detailed description is provided in the supplemental material.

### Virus capture assay

The SARS-CoV-2 virus capture assay was previously reported (75). A detailed description is provided in the supplemental material.

### Virus neutralization assay

Pseudoviral neutralization was performed as described (46, 69). A detailed description is provided in the supplemental material section.

### Western blot

At 48 h post-transfection, Spike-expressing HEK293T cells were washed in phosphate buffer saline (PBS) and then lysed with 1% Triton X-100 TNE lysis buffer [25 mM Tris (pH 7.5), 150 mM NaCl, 5 mM ethylenediaminetetraacetic acid (EDTA)] supplemented with protease inhibitors cocktail (ThermoFisher scientific). Cell lysates were then centrifuged to pellet cell debris, and cleared supernatants were transferred to new tubes. Laemmli buffer (Bio-Rad) with 5% B-mercaptoethanol (Bio-Rad) was then added to the cell lysates and subsequently heated at 95°C for 5 min. Cell lysates were then resolved by sodium dodecyl-sulfate polyacrylamide gel electrophoresis (SDS-PAGE) and transferred to nitrocellulose membranes. Membranes were blocked for 1 h at room temperature with blocking buffer (3% skim milk powder dissolved in tris-buffered saline supplemented with 0.1% Tween-20 [TBS-T]) and then probed with CV3-25 mAb (1 µg/mL) followed by horseradish peroxidase (HRP)-conjugated anti-human IgG (1:3,000, Invitrogen). HRP enzyme activity was determined after the addition of a 1:1 mix of Western Lightning oxidizing and luminol reagents (PerkinElmer Life Sciences, Waltham, MA, USA), and quantification of bands was performed using the ImageLab software (Bio-Rad).

### Cold inactivation

Pseudoviruses harboring the different S glycoproteins were produced as described in the supplemental material section, and small aliquots of each pseudovirus-containing supernatant were incubated on ice for different lengths of time (0, 6, 24, 48, and 72 h). Following incubation on ice, pseudoviruses were added in quadruplicate on HEK293T-ACE2 target cells which were seeded 24 h before infection at a density of 1 × 10^4^ cells/well in 96-well luminometer-compatible tissue culture plates (PerkinElmer, Waltham, MA, USA). Infectivity was measured as described above, with infectivity being relative to 0 h on ice. The inactivation half-maximal inhibitory time (*t*_1/2_) represents the time needed to inhibit 50% of the infection of HEK293T-ACE2 cells by pseudoviruses.

### Biolayer interferometry

Binding kinetics were performed using an Octet RED96e system, as described (55, 56). A detailed description is provided in the supplemental material.

### Molecular dynamics (MD) simulations

MD simulations of BA.2.86 Spike were performed using Nanoscale Molecular Dynamics (NAMD v2.14) (76) and the Chemistry at Harvard Macromolecular Mechanics (CHARMM) force field (77) using models of the Spike trimeric ectodomain based on the BA.2 structure in PDB entry 7XIX (78). For BA.2.86, three independent replica systems were generated, fully glycosylated, and fully solvated with three-site model (TIP3P) water using pestifer 1.2.9 (pypi.org/pestifer). Tertiary structural changes were monitored by measuring the distance between the center of mass of each RBD and the trimer center of mass. For BA.2.86, statistics on this distance are aggregated over all protomers within a trimer and over all trimer replicas.

### Lineage growth rate estimation

We computed lineage growth rate estimates from Global Initiative on Sharing All Influenza Data (GISAID) metadata (downloaded 8 January 2024) based on pangolin annotation (79). We exclusively considered sequences labeled as ‘Omicron’, originating from human hosts, and featuring fully specified deposition and collection dates. Following Dadonaite et al. (50), we required sequences to have a collection date within 150 days of deposition, we excluded sequences with collection dates falling outside of 3.5 times the interquartile range of the median, and only sequences from countries with more than 500 samples and from lineages with more than 200 samples were retained. For the estimation of clade growth rates for the remaining 1,170 lineages, we used a multinomial logistic regression model to analyze global lineage frequency data, accessible at the following repository: https://github.com/MurrellGroup/MultinomialLogisticGrowth/.

Notably, our data set includes analysis of emerging variants, such as JN.1, which differs from Dadonaite et al.’s results (50). In a comparative assessment of the 969 lineages analyzed by both methodologies, we obtained a near-perfect correlation with the recomputed estimates (Pearson correlation *r* = 0.9987639, *P*-value < 2.2e−16), validating our results. Reported growth estimate for BA.4/BA.5 represents an average value derived from these two distinct lineages.

### Statistical analysis

Statistical analyses were done using GraphPad Prism version 8.4.2 (GraphPad). Every dataset was tested for statistical normality, and this information was used to apply the appropriate (parametric or nonparametric) statistical analysis. *P*-values < 0.05 were considered significant; significance values are indicated as *P < 0.05, **P < 0.01, ***P < 0.001, ****P < 0.0001.

## RESULTS

### Spike characteristics of recent Omicron sublineages

The diversification of Omicron sublineages is mainly driven through accumulation of mutations in its Spike glycoprotein (19). Compared with BA.2, BA.2.75 possesses nine additional mutations in its Spike (Fig. 1A), conferring improved ACE2 binding and higher evasion from vaccine-elicited and monoclonal antibodies (mAbs). It was demonstrated that two mutations in the RBD, G446S and N460K, had a profound effect (23, 80–82). CH.1.1, one of the most frequently observed subvariant derived from BA.2.75, gained more attention in Asia and Europe (Fig. 2A) (22). Compared to BA.2.75, CH.1.1 has four additional mutations in its RBD, R346T, K444T, L452R, and F486S (Fig. 1A). These mutations have been reported to increase neutralization escape from polyclonal serum and mAbs (16, 22, 83, 84). Interestingly, L452R, which is also present in the Spike of BA.4, BA.5 and their progeny BQ.1.1, also improves RBD and Spike affinity to the ACE2 receptor (52, 85–87). BQ.1.1 harbors three additional antibody-escape mutations in its RBD compared to BA.4/BA.5: R346T, K444T, and N460K (14, 24, 53, 88, 89). While infections involving XBB were increasing due to its improved antibody evasion (16, 25, 89–91), XBB.1.5 quickly dominated over XBB and BQ.1.1 due to its improved ACE2 affinity over XBB (27–29) and improved immune escape compared to BQ.1.1 (92–94).

SARS-CoV-2 descendants of XBB.1 with the S:S486P mutation (Fig. 1A and 2A), also known to enhance ACE2 affinity (27), are predominantly circulating worldwide (30). Notably, XBB.1.16, harboring in its Spike the E180V, S486P, and T478R substitutions, showed a remarkable growth advantage compared to XBB.1.5 although both Spike glycoproteins possess similar characteristics in terms of infectivity and immune escape (95, 96). Predominant lineages possess a substitution at either or both positions S:L455F and S:F456L, mostly known as the ‘’FLip’’ mutations (L455F + F456L) (31, 97, 98). The ‘’FLip’’ mutations have been shown to act synergistically to further enhance ACE2-binding affinity and neutralization escape from plasma (97). Compared to XBB.1.5, FD.1.1 harbors the F456L substitution, EG.5.1 has the Q52H, and F456L substitutions while its sublineage named HK.3 also acquired the L455F mutation. DV.7.1, a sublineage of CH.1.1, also started spreading which may be due to the acquisition of the “FLip’’ mutations (Fig. 1A) (97, 99). BA.2.86, a highly divergent BA.2 subvariant, possesses mutations in its Spike enhancing its immune evasion and ACE2 binding compared to XBB.1.5, notably N450D, K356T, L452W, A484K, V483del, and V445H, with the addition of R403K being responsible for its enhanced ACE2 binding affinity (33, 100–103) (Fig. 1A and 2A). Lastly, the BA.2.86 subvariant JN.1 possesses an additional substitution at S:L455S, conferring improved immune evasion in spite of reduced ACE2 binding (30, 32, 35).

### Plasma recognition and neutralizing activity

To monitor the humoral responses against recent Omicron subvariants, we collected plasma samples from 18 donors (11 females and 7 males), with a total median age of 59 years (interquartile range: 51–64 years) (Table 1). Out of the 18 participants, 9 had detectable levels of anti-nucleocapsid antibody levels, suggesting a recent breakthrough infection. All participants received either the Moderna or Pfizer mRNA monovalent (WT) vaccines prior to the fifth dose. Plasma samples were collected 3–4 weeks after the 5th dose of mRNA vaccine with 13/18 receiving the Pfizer mRNA BA.4/5 bivalent vaccine, 2/18 receiving the Pfizer monovalent WT vaccine and 3 receiving the Moderna mRNA BA.1 bivalent vaccine (Table 1).

We first evaluated the capacity of plasma to recognize recent Omicron subvariants by transfecting HEK293T cells with plasmids encoding the full-length SARS-CoV-2 Spikes (52, 104). The CV3-25 monoclonal antibody (mAb) was used for the normalization of Spike expression levels across experimental conditions. CV3-25 is a Spike-binding mAb, specific against a conserved epitope shared among β-coronaviruses within the S2 subunit (70, 74, 105, 106). We observed that all tested Omicron subvariant Spikes were less efficiently recognized by plasma than the D614G Spike, with DV.7.1 (x3.5), HK.3 (x2.8), and JN.1 (x2.7) Spikes demonstrating the highest fold change in recognition by plasma (Fig. 1B). Interestingly, BA.2.86 (x2.0) was better recognized than CH.1.1 (x2.5), XBB.1.5 (x2.2), and EG.5.1 (x2.2). As expected, BA.4/5 (x1.6), BQ.1.1 (x1.8), and BA.2.75 (x1.9) had a decreased ability to evade recognition by plasma from vaccinated individuals compared to BA.2.75- and XBB-sublineages (Fig. 1B).

We next measured the capacity of plasma to neutralize pseudoviral particles harboring Spikes from emerging Omicron sublineages. To do this, we produced pseudoviral particles bearing the Spike glycoprotein from several omicron subvariants, as previously described (46, 47, 69, 107). As expected, all plasma strongly neutralized pseudoviral particles bearing the D614G Spike after the fifth dose of mRNA vaccine (Fig. 1C). In agreement with Spike recognition by plasma (Fig. 1B), most Omicron subvariants demonstrated significant neutralization escape, with the DV.7.1 (x66.8) and HK.3 (x57.1) Spikes being the most resistant to neutralization and the BA.2.75 (x1.4) and BA.4/5 (x.1.4) Spikes the most efficiently neutralized by plasma from vaccinated individuals. Interestingly, we observed a slight enhanced neutralization escape with BA.2.86 (x12.2) compared to most XBB sublineages, which was further pronounced with JN.1 (x18.0) (Fig. 1C).

### Neutralization susceptibility to CV3-25 and ACE2-Fc

As a control for our neutralization experiments, we first measured the susceptibility of the different variants to the S2-targeting CV3-25 mAb which recognizes a highly conserved epitope (70, 74, 105, 106). As expected, all subvariants tested were similarly susceptible to CV3-25 neutralization, with an IC_50_ in the range of 0.09–0.33 µg/mL (Fig. 2B). Given that recent Omicron subvariants have acquired mutations known to enhance ACE2 affinity, we next evaluated the ability of recombinant human ACE2-Fc to neutralize these subvariants using pseudoviral particles (72). We found that most subvariants had improved susceptibility to ACE2-Fc neutralization compared to D614G (Fig. 2C). However, XBB and XBB.1 were remarkedly less susceptible to ACE2-Fc neutralization compared to D614G. Interestingly, CH.1.1 and DV.7.1 were less sensitive to ACE2-Fc compared to their parental strain BA.2.75, although an improvement was seen for DV.7.1, possibly owing to the “FLip’’ mutations (Fig. 1A and 2C). As expected, XBB sublineages harboring the S:S486P were neutralized more efficiently than the parental XBB.1 strain, with EG.5.1 and HK.3 being the most susceptible (Fig. 1A, 2A and C). The additional mutations in BQ.1.1 decreased its susceptibility to ACE2-Fc-mediated neutralization compared to BA.4/5. Of note, among all the Omicron subvariants tested, BA.2.86 was the most susceptible to ACE2-Fc neutralization, with a 4.7× fold decrease in IC_50_ compared to D614G (Fig. 2C). The additional substitution in JN.1 considerably reduced its sensitivity to ACE2-Fc neutralization compared to BA.2.86.

### Impact of temperature on the RBD-ACE2 interaction

**Fig 3:**
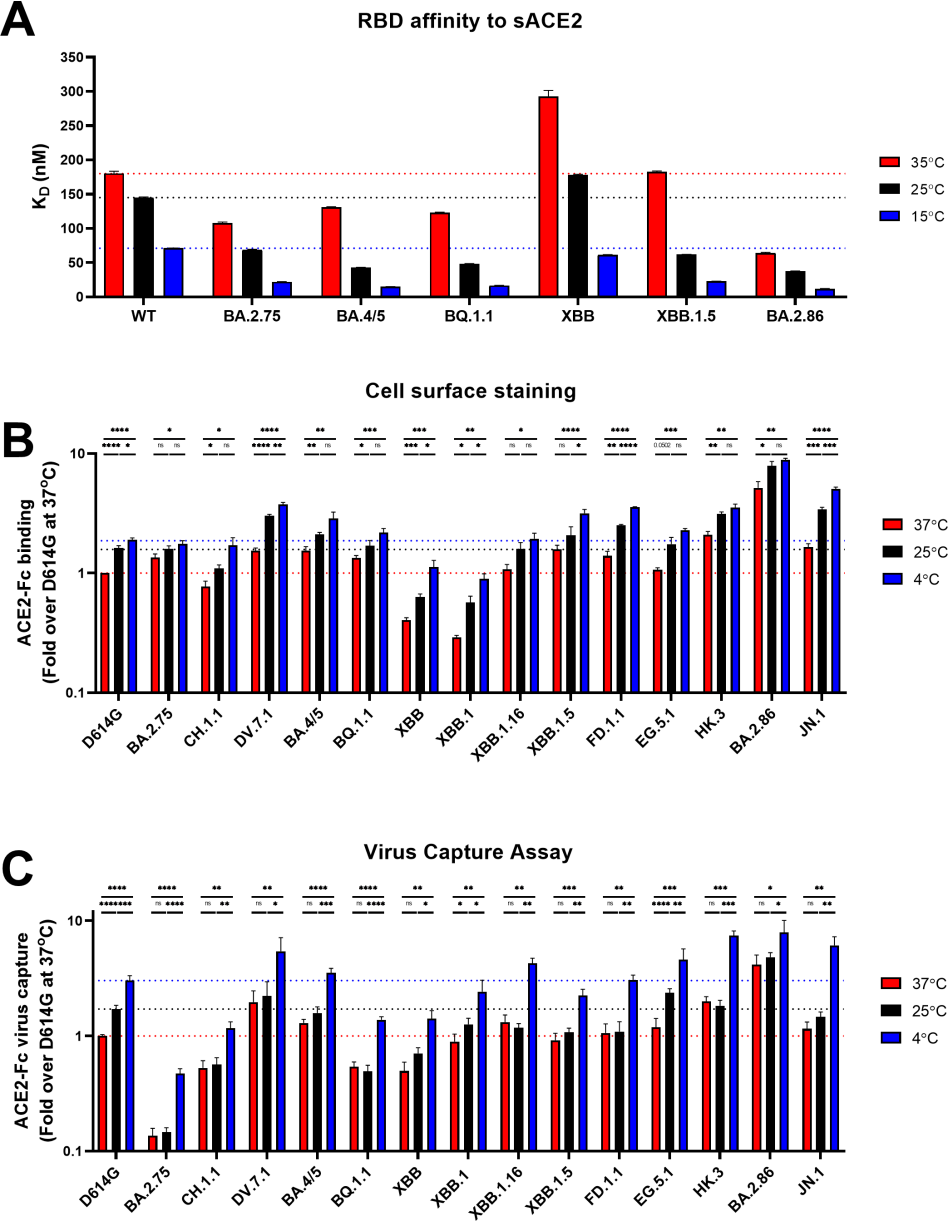
Low temperatures enhance Omicron subvariants RBD affinity and Spike interaction with ACE2. (**A**) Binding kinetics between RBD_WT_ and several RBD_Omicron_ with sACE2 were assessed by BLI at different temperatures. Biosensors loaded with RBD proteins were soaked in two-fold dilution series of sACE2 (500–31.25 nM) at different temperatures (35°C, 25°C, or 15°C) with the affinity constants (*K*_D_) being represented along with the technical error. Affinity values obtained at different temperatures were calculated using a 1:1 binding model. (**B and C**) Cell surface staining of Spike-expressing HEK293T cells and virus capture assay of WT (D614G) and Omicron subvariant Spikes at 37°C (red), 25°C (black), and 4°C (blue). (**B**) ACE2-Fc recognition is presented as a ratio of ACE2 binding over D614G Spike obtained at 37°C. (**C**) Pseudoviruses encoding the luciferase gene (Luc+) and expressing SARS-CoV-2 Spikes were tested for viral capture by ACE2-Fc at the respective temperatures. Relative light units (RLU) obtained using ACE2-Fc were normalized to the signal obtained with the temperature-independent CV3-25 mAb and presented as a ratio of ACE2 capture to D614G obtained at 37°C. (**B and C**) These results represent at least three independent experiments showing means ± SEM. Statistical significance was tested using unpaired *T* tests (**B and C**) (*P < 0.05; **P < 0.01; ***P < 0.001; ****P < 0.0001; ns, non-significant).

Previous studies showed that SARS-CoV-2 Spike interaction with ACE2 can be modulated by temperature, with lower temperatures enhancing RBD affinity for ACE2 (55, 56). To evaluate whether this property was conserved with these new Omicron subvariants, we measured the RBD affinity to soluble ACE2 (sACE2) by performing biolayer interferometry (BLI) experiments at different temperatures (35°C, 25°C, and 15°C). In agreement with previous observations (52, 55), we found that cold temperatures enhanced the affinity of the RBDs tested with ACE2 (Fig. 3A). The higher affinity observed at colder temperatures for the different Omicron subvariants RBDs compared to wild-type (WT) RBD was mainly explained by a drastic decrease in the off rate (Fig. S1A). Interestingly, except for RBD_XBB_, the RBD of Omicron subvariants had similar or better affinity for ACE2 at 25°C compared to RBD_WT_ at 15°C, and these differences were further enhanced for all Omicron subvariants at 15°C. The RBD_BA.4/5_ and RBD_BQ.1.1_ affinity was relatively similar at all temperature tested. In agreement with our ACE2-Fc neutralization results, RBD_BA.2.86_ had the highest affinity for sACE2 among the different Omicron subvariants, and this remained true for all temperature tested.

Moreover, the RBD of BA.2.75, BA.4/5, BQ.1.1, and BA.2.86 bound better to ACE2 compared to RBD_WT_ at the three temperatures tested (Fig. 3A). Of note, the affinity of RBD_XBB_ was remarkedly lower compared to RBD_WT_, but this was restored with the acquisition of S:S486P as shown with RBD_XBB.1.5_, thus corroborating the ACE2-Fc neutralization results observed for XBB/XBB.1 and XBB.1.5 (Fig. 2C).

### Impact of temperature on Spike-ACE2 interaction

We next measured the capacity of ACE2-Fc to bind the full-length Omicron subvariants Spikes expressed at the surface of transfected HEK293T cells using a well-established flow cytometry assay (52, 55, 56, 72) (Fig. 3B). We used the temperature-independent S2-targeting CV3-25 mAb as a control for each temperature (55, 56, 74) (Fig. S1B). Lower temperatures have been shown to modulate the capacity of the trimeric Spike to interact with ACE2 by favoring the adoption of the RBD “’up’’ conformation, required for ACE2 binding (45, 55, 56). To extend these results on recent Omicron subvariants, we evaluated ACE2-Fc binding with Spike-expressing cells after incubation at 37°C, 25°C, and 4°C (Fig. 3B). In agreement with the results obtained by BLI (Fig. 3A; Fig. S1C), we observed a gradual increase in ACE2-Fc binding concomitant with the temperature decrease for all Spikes tested. Of note, DV.7.1, BA.4/5, XBB.1.5, FD.1.1, HK.3, BA.2.86, and JN.1 showed better binding at 25°C than D614G at 4°C, and these differences were further enhanced at 4°C. Interestingly, HK.3 and BA.2.86 showed enhanced binding at 37°C compared to D614G binding at 4°C. As expected from previous reports showing higher affinity for RBD interaction (32, 33), BA.2.86 presented the highest binding capacity at all temperatures tested, showing more than 8.8-fold improvement at 4°C compared to D614G at 37°C. As expected, JN.1 showed reduced ACE2-Fc binding compared to its parental lineage BA.2.86. In addition, DV.7.1, HK.3, and BA.2.86 had the highest ACE2-Fc-binding capacity at 37°C, with BA.2.86 demonstrating the greatest binding (Fig. 3B). The acquisition of S:S486P improved ACE2-Fc binding for XBB.1.16, XBB.1.5, FD.1.1, and EG.5.1 compared to XBB/XBB.1, independently of the temperature.

We next evaluated the impact of temperature on ACE2 interaction with Spike expressed at the surface of pseudoviral particles. To investigate this, we used a previously described virus capture assay (55, 56, 75, 108). Briefly, we produced pseudoviral particles bearing the different Omicron subvariant Spikes and evaluated their ability to interact with ACE2-Fc on ELISA plates. Pseudoviral particles were pre-incubated at different temperatures (37°C, 25°C, and 4°C). In agreement with enhanced RBD affinity and Spike interaction with ACE2 at lower temperatures (Fig. 3A and B), we observed a stepwise increase in viral capture at colder temperatures for all Spikes tested although the increase at 25°C was modest for most Spikes (Fig. 3C). These results significantly correlated with the RBD affinity and cell-based binding assay at different temperatures (Fig. S1C and D). Interestingly, we also saw an improved binding for DV.7.1, HK.3, and BA.2.86 at 37°C, which was higher than that of D614G at 25°C, with BA.2.86 showing the greatest capture by ACE2 at all respective temperatures tested (Fig. 3C). On the other hand, JN.1 showed a marked capture at 4°C despite having reduced virus capture at 37°C and 25°C. Altogether, our findings demonstrate that Spike-ACE2 interaction of recent Omicron subvariant is modulated by temperature independently of whether the Spike is expressed on pseudoviral particles or at the cell surface, likely explained through higher RBD affinity.

Of note, at 37°C most Omicron subvariants bound ACE2-Fc better than D614G (Fig. 3C). We also noticed a decrease in ACE2 capture for BQ.1.1 and JN.1 compared to BA.4/5 and BA.2.86, respectively, which corroborated the results obtained by ACE2-Fc neutralization and binding (Fig. 2B and 3B). Interestingly, XBB.1 but not XBB had a similar capture by ACE2-Fc compared to D614G. As expected, lineages harboring the S:S486P substitution had improved capture by ACE2-Fc, and this was more noticeable for XBB.1.16 (Fig. 3C).

### Impact of temperature on ACE2-binding cooperativity

Since Spike interaction with ACE2 requires RBD to be in the “up’’ conformation (39, 109, 110), we wondered whether recent Omicron subvariants had an improved propensity to adopt the “up'’ conformation at lower temperatures as previously observed for SARS-CoV-2 D614G and early omicron strains (55, 56, 111). To investigate this, we calculated the Hill coefficient (h), which is a measure indicating the degree of binding cooperativity between protomers of a multimeric protein with its ligand (112, 113). Briefly, we transfected HEK293T cells with the full-length Spikes of recent Omicron subvariants and incubated these cells with increasing concentrations of monomeric soluble ACE2 as described previously (55, 56). The Hill coefficient was calculated based on the steepness of the dose-response curves, as described in the Materials and Methods.

**Fig 4:**
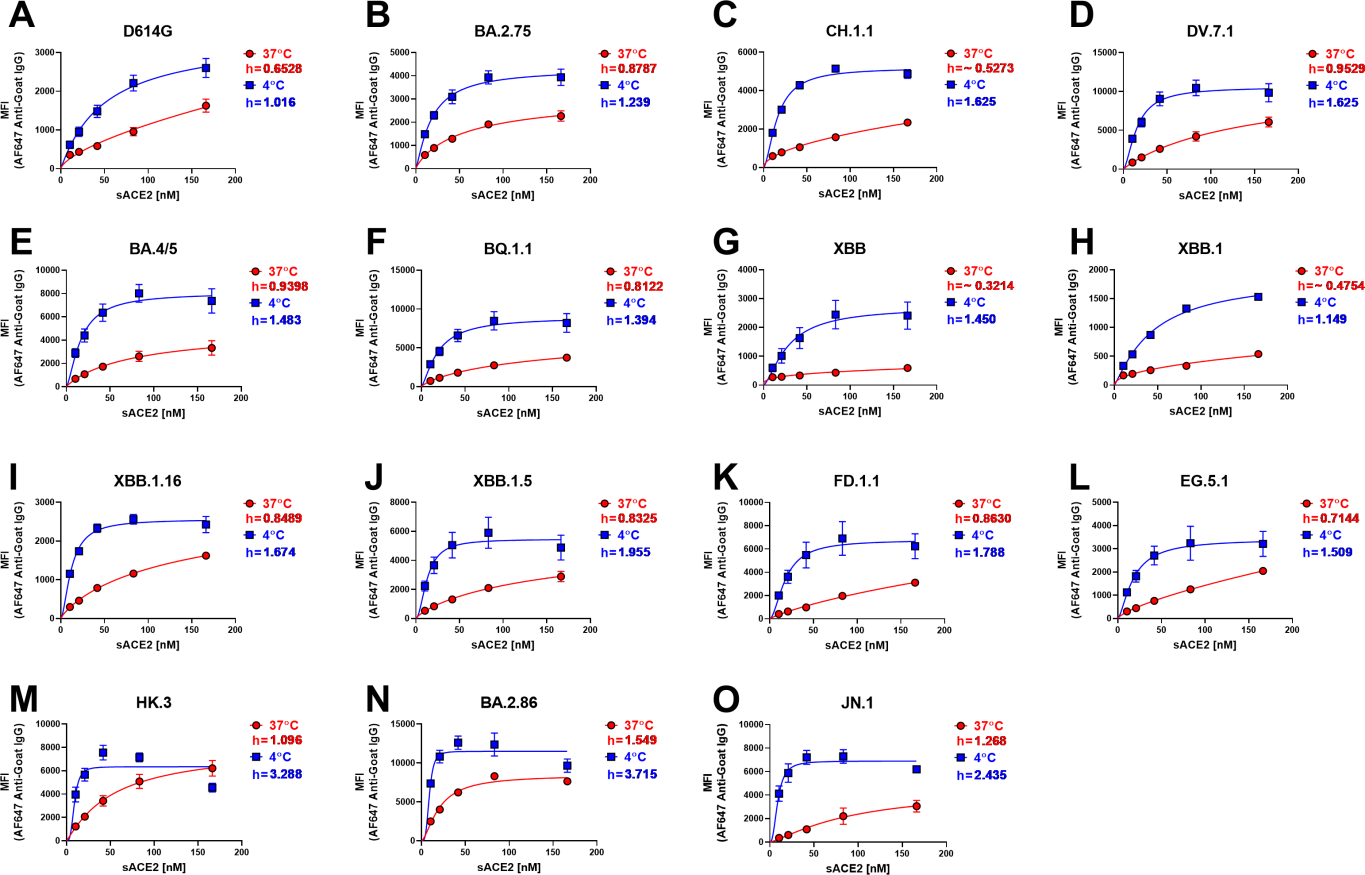
Omicron subvariant Spikes “open-up” at low temperature. The binding of sACE2 to the Spike of D614G and several Omicron subvariants expressed at the surface of HEK293T cells was measured by flow cytometry. Increasing concentrations of sACE2 were incubated with Spike-expressing cells at 37°C (red) or 4°C (blue). Means ± SEM derived from at least three independent experiments are shown. Hill coefficients were calculated using the GraphPad software.

Consistent with previous observations, the Hill coefficient at low temperature (4°C) was greater than at 37°C for all Spikes tested, confirming that low temperatures facilitate ACE2-induced Spike opening (Fig. 4) (55, 56). Compared to D614G, all Omicron subvariants showed enhanced cooperativity at 4°C, with HK.3, BA.2.86, and JN.1 having the highest hill coefficient (Fig. 4M through O). We also calculated the Hill coefficient’s fold change between 4°C and 37°C to compare the impact of low temperatures on Spike cooperativity between variants. We observed that the Hill coefficient’s fold change for nearly all Omicron subvariants was higher than that of D614G, and this was more evident for XBB sublineages (Fig. S2).

We also observed that at 37°C, most Omicron subvariants had a higher Hill coefficient than D614G (Fig. 4), except for CH.1.1, XBB, and XBB.1 (Fig. 4A, C, G and H). Consistent with the ACE2-Fc binding at the cell surface and viral particles (Fig. 3B and C), XBB.1.16, XBB.1.5, FD.1.1, and EG.5.1 demonstrated a marked improvement in promoter cooperativity compared to XBB.1, likely due to the S:S486P substitution (Fig. 4G through L). Aligned with their improved ACE2-Fc binding, DV.7.1, HK.3, and BA.2.86 showed the best cooperativity at 37°C among the Omicron subvariants tested (Fig. 4D, M and N). Interestingly, despite lower ACE2-Fc binding at the surface of transfected cells and pseudoviruses, JN.1 shows high levels of cooperativity at 37°C (Fig. 4O). These results suggest that Omicron subvariants acquired mutations improving their Spike opening.

### Spike processing is associated with susceptibility to cold inactivation

**Fig 5:**
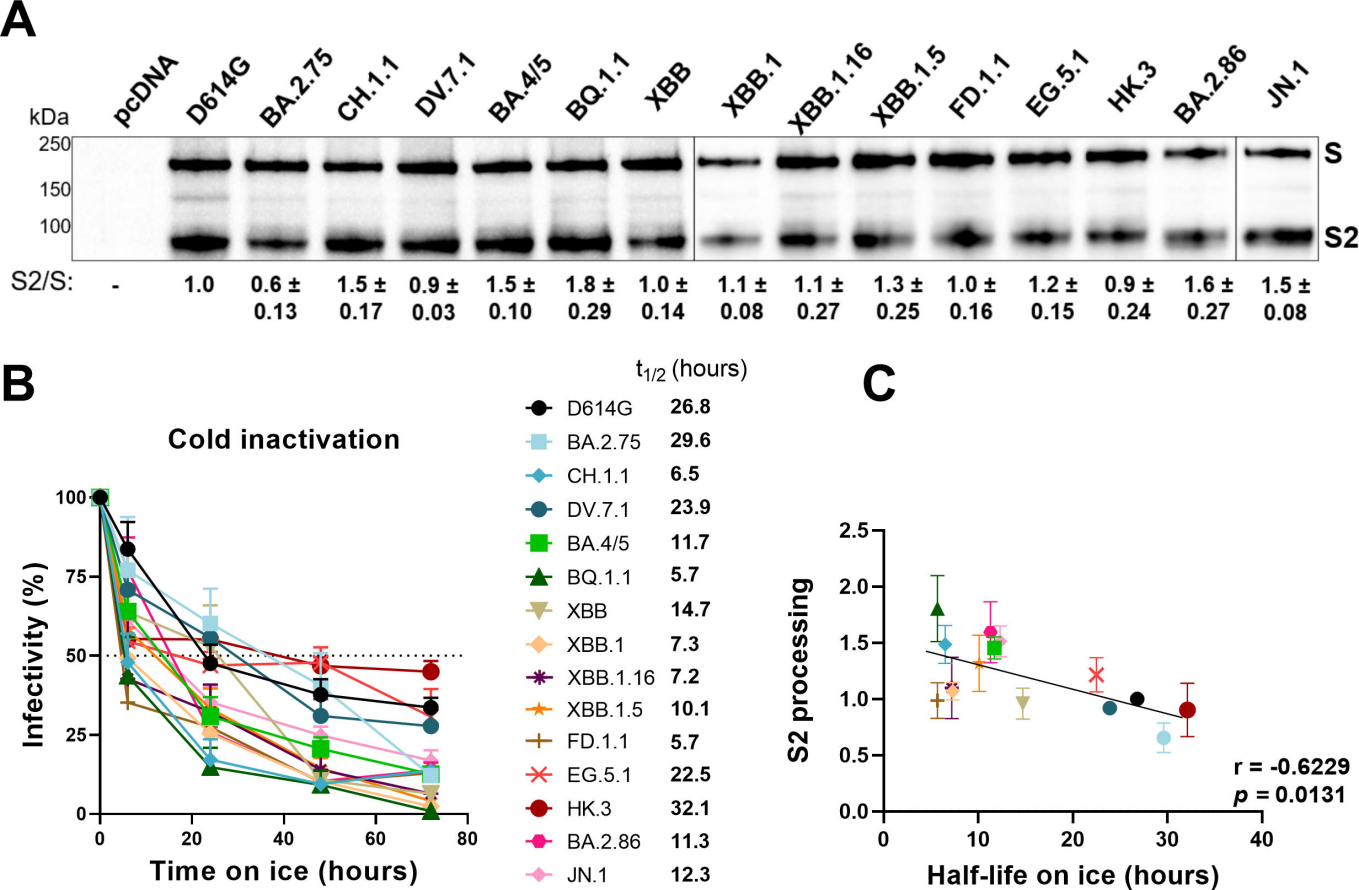
Omicron subvariants’ Spike processing and susceptibility to cold inactivation. (**A**) HEK293T cells were transfected with several Omicron subvariants Spikes, which were subsequently lysed and analyzed by Western blot using the anti-S2 CV3-25 monoclonal antibody. The ratio of processed S2 over unprocessed S is shown (S2/S), with values being normalized to D614G for each experiment. Values represent the mean of three to four independent biological replicates, with the SEM being shown. (**B**) To evaluate the susceptibility of Omicron subvariants to cold inactivation, pseudoviral particles bearing the several Spikes were incubated on ice for 0, 6, 24, 48, and 72 h, followed by infection of HEK293T-ACE2 cells. These results represent at least three independent experiments showing means ± SEM. The time required to reduce infection by 50% (*t*_1/2_) was determined using a normalized nonlinear regression using GraphPad Prism software. (**C**) Pearson rank correlations between the S2/S ratio with the half-life on ice.

It was observed that SARS-CoV-2 Spike susceptibility to cold inactivation may reflect the propensity to sample more “open’’ Spike conformations, thus facilitating conformational transitions (66). Interestingly, it has been demonstrated that Omicron BA.1 S1/S2 Spike processing is reduced compared to D614G and Delta, and that concomitantly, BA.1 Spike is more susceptible to cold inactivation (66). To test the hypothesis that Spike cleavage could be associated with susceptibility to cold inactivation, we first measured Spike processing at the cell surface (Fig. 5A). HEK293T cells were transfected with the Omicron subvariants' Spikes and subsequently examined by Western blotting using the anti-S2 CV3-25 mAb (Fig. 5A). Compared with D614G, most Omicron subvariants exhibited similar or higher cleavage of S to S2 (S2/S ratio), with the exception of BA.2.75 whose processing was decreased. Interestingly, CH.1.1 and BQ.1.1 had an enhanced processing compared to their respective parental lineage. Among the XBB.1 sublineages, XBB.1.5 and EG.5.1 exhibited the highest level of processing, with HK.3 showing reduced processing. BA.2.86 and JN.1 presented improved processing compared to D614G and XBB.1 sublineages.

To investigate the role of S2 processing in the susceptibility of recent Omicron subvariants to cold inactivation, we next evaluated the effect of cold (0°C) on the infectivity of pseudoviral particles (66–68, 114). Briefly, pseudoviral particles bearing the different Omicron subvariants' Spikes were incubated on ice for various amounts of time and their capacity to infect HEK293T-ACE2 cells was subsequently measured (Fig. 5B). Interestingly, except for BA.2.75 and HK.3, all Omicron subvariants were more susceptible to cold inactivation compared to D614G, with FD.1.1 and BQ.1.1 being the most susceptible. Compared to CH.1.1 and EG.5.1, the acquisition of the “FLip’’ mutations seemed to improve the stability of DV.7.1 and HK.3 Spikes, respectively. We found a negative correlation between S2 processing (S2/S) and the half-life on ice (*t*_1/2_), suggesting that enhanced processing is associated with higher susceptibility to cold inactivation (Fig. 5C).

### Molecular dynamics simulations of BA.2.86 Spike are consistent with more open trimers at lower temperatures

**Fig 6:**
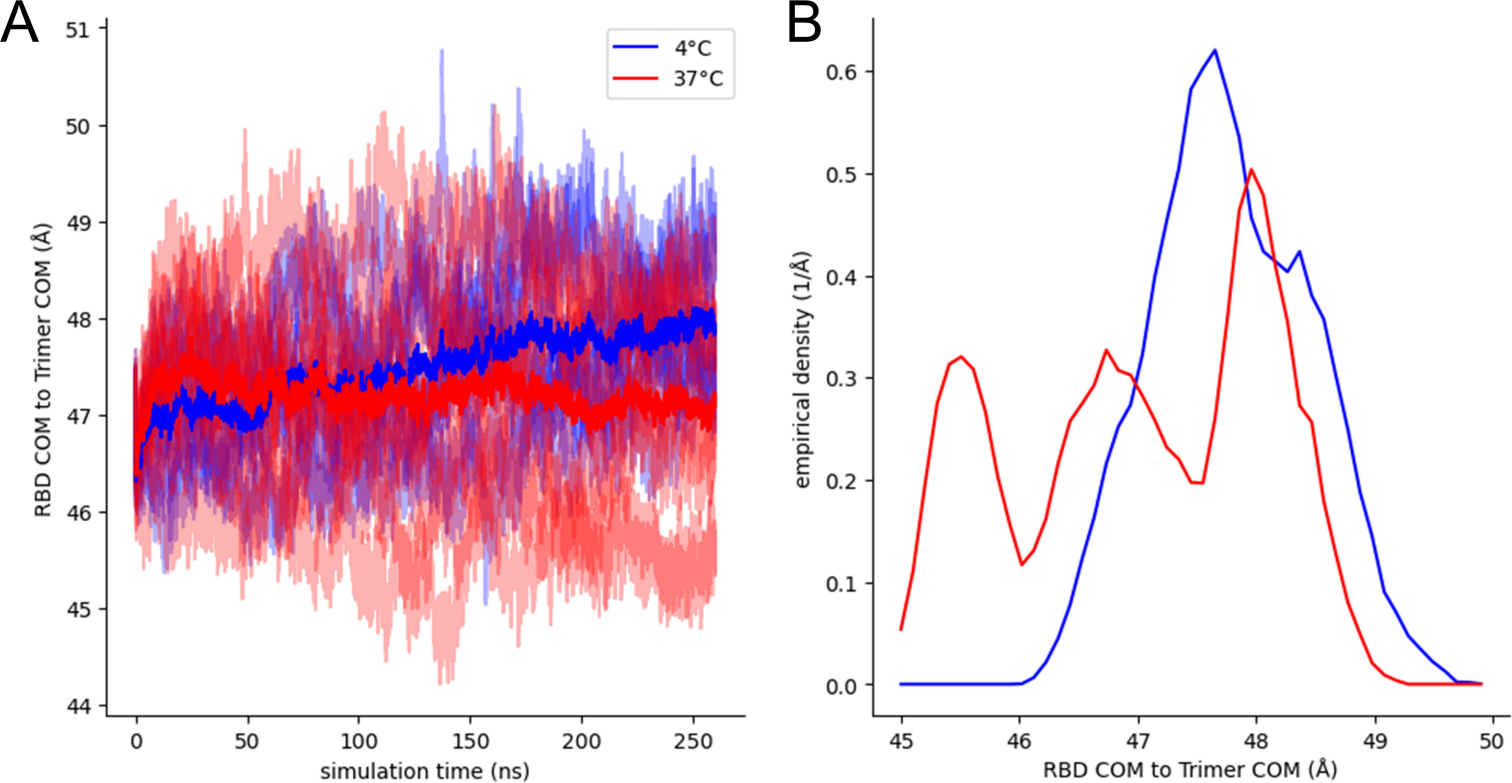
MD simulations of BA.2.86 Spike are consistent with more open trimers at lower temperatures. (**A**) Traces of the distances between RBD and trimer centers of mass (COM) from three replicas each of all-atom, fully glycosylated, and solvated MD simulations of the closed, BA.2.86 SARS-CoV-2 S trimers at 4°C (blues) and 37°C (reds) with data set averages shown in heavy traces. Each set has nine distinct traces. (**B**) Empirical density distributions of these distances sampled after 150 ns of simulation time.

To corroborate our findings that lower temperatures improve the propensity of Omicron subvariants RBD to adopt the “up’’ conformation, all-atom, explicit-solvent MD simulations were performed on three independent replicas of BA.2.86 homology models based on the closed BA.2 spike structure at both 4°C and 37°C (Fig. 6). The degree of opening of the RBDs was assessed by measuring the instantaneous distance between RBD center of mass and the center of mass of the full trimer, as described (55). Plots of this distance vs simulation time and their empirical distributions are shown in Fig. 6B. The BA.2.86 systems required more than 150 ns of equilibration, after which the colder spikes showed distances of about 48 Å and warmer spikes about 47 Å. The Wuhan strain spike considered in our earlier work (55) showed distances of about 46 Å at 4°C and 45 Å at 37°C, which is a similar sensitivity to temperature but with overall more closed-down RBDs relative to BA.2.86. The equilibrium empirical distributions of these distances show that the warmer spikes sample three distinct states, while the colder spikes are more uniformly in a single, more open state (Fig. 6).

### ACE2 interaction at the surface of viral particles is associated with viral growth rates

**Fig 7:**
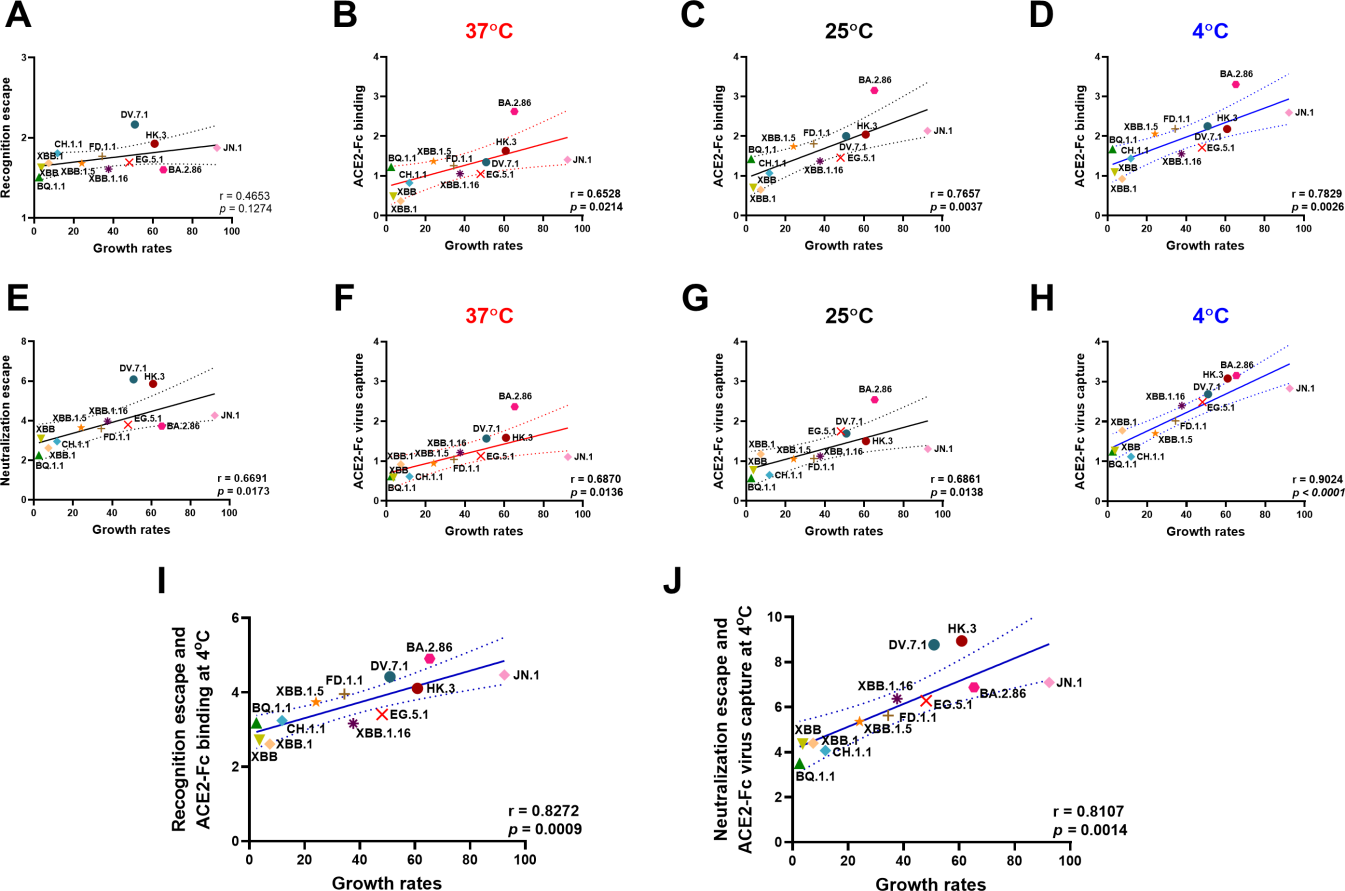
Associations between growth rates of several Omicron subvariants and immune escape or ACE2 interaction at different temperatures. Correlation of growth rates with plasma recognition (**A**) or ACE2-Fc binding to Spikes presented at the cell surface at 37°C (**B**), 25°C (**C**), and 4°C (**D**). Correlation of growth rates with plasma neutralization (**E**) or ACE2-Fc virus capture at 37°C (**F**), 25°C (**G**), and 4°C (**H**). Plasma recognition and neutralization escape, ACE2-Fc binding, and ACE2-Fc virus capture were normalized to the values obtained for D614G at 37°C and subsequently log_2_ transformed. Panels I and J represent correlations using the combination of plasma recognition or neutralization escape with ACE2-Fc binding at the surface of cells or viral particles. (A–J) Pearson rank correlations are shown. Panels A, E, I, and J refer to data shown in Fig. 1. Panels B–D, F–H, and I and J refer to data shown in Fig. 3 (*P < 0.05; **P < 0.01; ***P < 0.001; ****P < 0.0001).

SARS-CoV-2 viral transmission is a complex multifactorial process where immune pressure, tropism, and affinity for its receptor affect the outcome (5). We investigated whether Spike recognition, either at the surface of cells or viral particles, as well as ACE2 interaction was associated with viral growth rates. To do this, we calculated the growth rates of SARS-CoV-2 Omicron sublineages following the methodology developed by Dadonaite et al. (50) to incorporate recently emerged Omicron subvariants as of 8 January 2024. Interestingly, plasma neutralization of pseudoviral particles bearing emerging Omicron subvariants' Spikes, but not Spike recognition at the cell surface, was significantly associated with viral growth rates (Fig. 7A and E). Intriguingly, the capacity of the different Spikes to interact with ACE2, particularly at low temperature, was significantly associated with the growth rates (Fig. 7B through D and F through H). More pronounced associations were observed when the Spike was expressed at the surface of viral particles rather than at the cell surface (Fig. 7D and H). Of note, the combination of both recognition escape or neutralization escape with ACE2-Fc binding at low temperatures correlated well with viral growth rates (Fig. 7I and J). Collectively, our results suggest that antibody escape and ACE2 interaction, particularly at low temperatures, influence viral transmission.

## DISCUSSION

**Fig 8:**
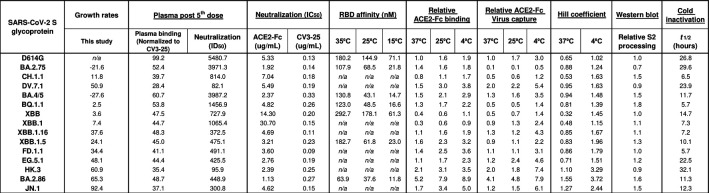
Omicron subvariant Spikes characteristics. Summarized data from Fig. 1 to 5 and 7 are shown. n/a = not available.

The continued evolution of SARS-CoV-2 requires constant monitoring of its new variants (21). Current circulating strains are derived from the Omicron variant, with each newly emerging subvariant showing improved transmission, which can mostly be explained by their enhanced antibody escape and ACE2 binding affinity (54, 115). To determine if other Spike characteristics are involved in viral growth rates, we characterized the functional properties of recent Omicron subvariant Spikes (Fig. 8), including their capacity to evade humoral responses.

We observed significant differences in the capacity of individuals receiving a fifth dose of bivalent (BA.1 or BA.4/5) mRNA vaccine to recognize and neutralize recent Omicron subvariants (Fig. 1B and C). The evolutions of CH.1.1 to DV.7.1, EG.5.1 to HK.3, and BA.2.86 to JN.1 led to an improvement in their capacity to evade humoral responses, which could likely be explained by the acquisition of mutations on either S:L455 or S:F456 (30–32, 97, 98). Consistent with other reports, we found that BA.2.86 possesses slightly better escape from plasma-mediated neutralization compared to XBB.1.5, and this was further pronounced with JN.1 (30, 32, 35, 98, 100, 102, 103). These results demonstrate that the high levels of preexisting immune pressure against SARS-CoV-2 Spike glycoprotein are driving its evolution and transmission (48).

By using various approaches, we characterized the ACE2-binding properties of recent Omicron subvariants Spikes. We found that among all Omicron subvariants tested, BA.2.86 was the most susceptible to ACE2-Fc neutralization and exhibited the highest RBD affinity, protomer cooperativity, and ACE2 binding at the surface of transfected cells and viral particles (Fig. 2 to 4). These results are in line with previous studies demonstrating the marked improvement in ACE2 binding affinity and explained notably through the acquisition of the S:R403K mutation (33, 101). Furthermore, this increased binding affinity could also be linked to the intrinsic charge properties of the region of ACE2 targeted by the Omicron RBD, which are negatively and positively charged, respectively (116–118). Notably, the additional positive charges within the RBD associated with mutations V445H, N460K, N481K, and A484K might contribute to its improved ACE2 binding (101). Recently, a BA.2.86 sublineage, JN.1, has shown improved growth rate due to one substitution S:L455S, improving its neutralization escape at the expense of ACE2 binding (32). In this study, we confirm these results by which JN.1 possesses improved immune escape concomitant with its lower ACE2 binding. This further demonstrates the delicate balance existing between neutralization escape and ACE2 affinity in SARS-CoV-2 transmission (5).

It has been suggested that the optimal air temperature for SARS-CoV-2 transmission ranges from 5°C to 15°C (57, 58). Within upper airways, lower temperature creates a gradient of temperature from the nasal cavity to the trachea, where it reaches around 33°C (119–122). We previously found that lower temperatures improve RBD-ACE2 interaction and enhance the Spike propensity to sample the “up’’ conformation leading to higher ACE2 binding, fusogenicity, and viral replication (52, 55, 56). By priming SARS-CoV-2 Spike, lower temperatures could enhance its ability to bind ACE2 in the upper airways, favoring the initial adsorption (61). In this study, we found that all Omicron subvariants Spikes tested remained sensitive to the impact of low temperatures, further enhancing their protomer cooperativity and ACE2 binding at the surface of transfected cells and viral particles (Fig. 3 and 4). More importantly, we found that most Omicron subvariants reached similar levels of binding at 25°C than that of D614G at 4°C at the cell surface and with the RBD, suggesting a lesser reliability on cold temperatures for their improved ACE2 binding. This impact was most notable with DV.7.1, HK.3, and BA.2.86. While multiple factors are involved in SARS-CoV-2 transmission, several lines of evidence demonstrated an association between the climate and increased disease transmission, with lower temperature and humidity being associated with higher COVID-19 incidence (62, 63, 123–129). Whether this is due to ACE2 binding at lower temperatures remains to be determined. Notably, the rapid spread of BA.2.86 and expansion of its sublineage JN.1 in North America and in “colder’’ Northern European countries such as the United Kingdom, Denmark, Sweden, Iceland, and France raises the intriguing possibility that lower temperatures could play a role in transmission through improved ACE2 binding (30, 32, 33, 100, 130, 131).

The Spike glycoprotein requires the adoption of the RBD “up’’ conformation to interact with its receptor ACE2 (44, 45, 132). One parameter affecting the propensity to adopt the “up’’ conformation, and thus ACE2 interaction, is the degree of inter-protomer cooperativity upon ACE2 binding within Spike trimers (72, 133). We and others have demonstrated that SARS-CoV-2 Spike from early variants such as Alpha, Delta, and Omicron (BA.1 and BA.4/5) have an improved inter-protomer cooperativity compared to D614G, which was further enhanced at low temperatures (55, 56, 133). Here, we show that Omicron evolution continues to follow this trend. We observe a marked improvement in cooperativity among recently emerging Omicron subvariants, with DV.7.1, HK.3, BA.2.86, and JN.1 showing the highest levels of cooperativity at 37°C compared to D614G, with these differences being further pronounced at low temperatures (Fig. 4). Interestingly, the convergent evolution of emerging Omicron sublineages harboring mutations at either S:L455 or S:F456 illustrates the importance of these residues in transmission (30, 97). Our results show that the acquisition of the “FLip’’ mutations strongly enhanced inter-protomer cooperativity, concomitant with higher ACE2 binding, with DV.7.1 and HK.3 showing a remarked improvement compared to their respective parental lineages (31, 97). Thus, the ongoing surveillance of emerging SARS-CoV-2 variants is most likely due to their concomitant improvement in neutralization escape, enhanced protomer cooperativity, and increased ACE2 binding, which could inform future COVID-19 vaccine design.

We also observed differences in S cleavage to S2 of cells expressing emerging Omicron subvariant Spikes (Fig. 5A). In agreement with previous reports, we found that BQ.1.1 and CH.1.1 were processed more efficiently than their respective parental lineages, which could impact their fusogenicity and intrinsic viral pathogenicity (24, 94, 134). We also found that BA.2.86 and JN.1 were more processed than XBB.1.5 and EG.5.1, likely explained by the S:P681R mutation known to enhance Spike processing, fusogenicity, and pathogenicity (33, 41, 134, 135). Whether this enhanced processing will affect pathogenicity in humans remains to be known.

We also found a correlation between S2 processing and susceptibility to cold inactivation (Fig. 5). In contrast to the early Omicron BA.1, recent Omicron subvariants possess enhanced processing while remaining remarkedly sensitive to cold inactivation (66). Interestingly, it was found that susceptibility to cold inactivation may reflect the propensity to sample more “open’’ Spike conformations, thus facilitating conformational transitions (66, 68). As such, cold inactivation might be a helpful tool to determine which variants sample more “open’’ conformations which may lead to better viral replication. Our results suggest that, at least for Omicron subvariants, S2 processing could modulate Spike conformation to adopt more “open’’ conformations, rendering them more susceptible to cold inactivation. Interestingly, DV.7.1 and HK.3, harboring the “Flip’’ mutations, had less S2 processing and were less sensitive to cold inactivation compared to their parental lineages, suggesting that “Flip’’ mutations might improve stability through decreased processing, while also strongly enhancing ACE2 binding and the effect of low temperatures. Of note, EG.5.1 was more resistant to cold inactivation than FD.1.1 and XBB.1.5, which could possibly be attributed to its S:Q52H mutation. Interestingly, it was found that EG.5.1 had an increased transmissibility and altered tropism from that of XBB.1.5 (136). Whether this is linked to its higher susceptibility to ACE2-Fc neutralization and improved stability remains to be established.

Finally, we also observed that plasma-mediated recognition, neutralization, and ACE2-Fc binding at the surface of cells or pseudoviral particles was associated with emerging Omicron subvariants growth rate (Fig. 7). The combination of both escape from plasma and ACE2 binding at low temperatures enhanced these associations. These observations further support that ACE2 interaction and immune escape is associated with SARS-CoV-2 transmission and evolution (54, 115). Intriguingly, we observed a higher correlation coefficient for ACE2 binding and virus capture with growth rates at lower temperatures, thus suggesting that temperature modulation of Spike-ACE2 interaction plays a role in viral transmission.

The continued evolution of SARS-CoV-2 requires constant monitoring of its ability to evade immune responses elicited by previous infections and/or vaccination. Our results suggest that the capacity of new emerging variants to interact with ACE2, particularly at low temperatures, is another parameter that deserves to be closely monitored. The growth advantage of XBB.1.5 and its sublineages compared to XBB demonstrate the importance of monitoring ACE2 interaction (28, 29, 40, 44, 110, 115). Although the exact mechanisms through which temperature affects SARS-CoV-2 transmission remain unclear, our findings indicate that Omicron subvariants have undergone mutations that enhance resistance to neutralization by plasma, improve S processing, and increase affinity for ACE2 at both low and high temperatures. Of note, we found that measurement of Spike-ACE2 interaction of viral particles at low temperatures is strongly associated with Omicron subvariants growth rates. Such measures can readily be performed upon the emergence of new variants and could help define which variants have the potential to rapidly expand. In summary, our study underscores the necessity for ongoing surveillance of emerging subvariants and their characteristic mutations, as this information is likely to inform which variants have the potential to become predominant and inform the development of vaccines and other interventions.

## Data Availability

Data and reagents are available upon request.

## SUPPLEMENTAL MATERIAL

Supplemental Figures mbio.00907-24-s0001.pdf Figures S1 and S2.

Supplemental Text mbio.00907-24-s0002.docx Supplemental methods.

ASM does not own the copyrights to Supplemental Material that may be linked to, or accessed through, an article. The authors have granted ASM a non-exclusive, world-wide license to publish the Supplemental Material files. Please contact the corresponding author directly for reuse.
