# Supplementary material for: Temperature-dependent Spike-ACE2 interaction of Omicron subvariants is associated with viral transmission
Source: mBio. 2024 Jul 2;15(8):e00907-24. doi: 10.1128/mbio.00907-24 (PMC11323525; doi:10.1128/mbio.00907-24)
Supplement: Supplemental Figures — Figures S1 and S2. [file mbio.00907-24-s0001.pdf]

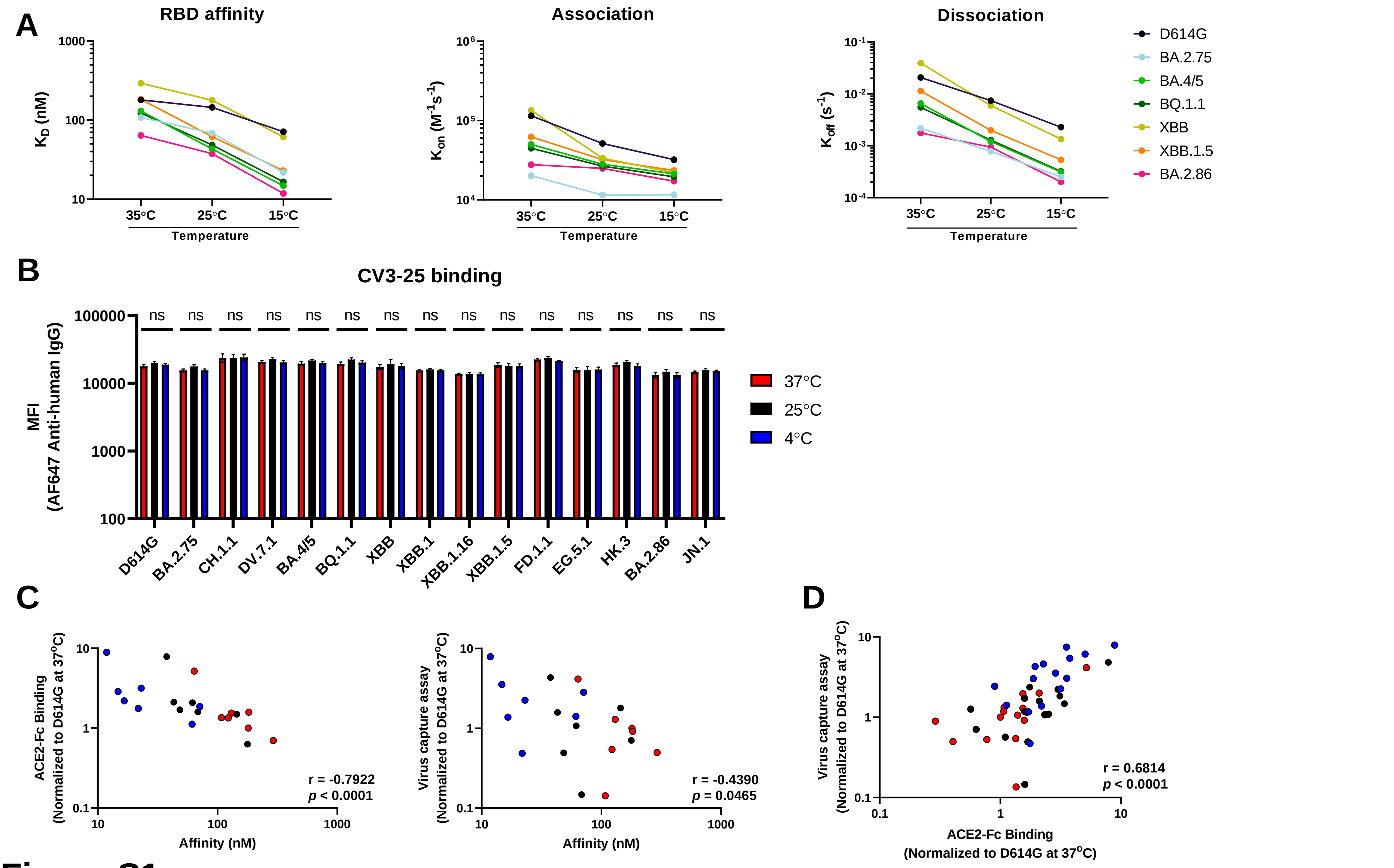

Figure S1

RBD binding kinetics, CV3-25 mAb binding and correlations among techniques at different temperatures

(A) Binding kinetics between several RBD (WT or Omicron subvariants) and sACE2 assessed by biolayer interferometry at different temperatures. Graphs represent the affinity constants (KD), on rates (Kon), and off rates (Koff) values obtained at different temperatures and calculated using a 1:1 binding model. (B) Cell surface staining of HEK293T cells expressing full length SARS-CoV-2 Spike glycoproteins from indicated variants (D614G and Omicron subvariants). Temperature-independent CV3-25 mAb is used to quantify the amount of Spike expressed on the cell surface. The graph presents the median fluorescence intensities (MFI). Error bars indicate means ± SEM. These results were obtained in at least three independent experiments. Statistical significance was tested using Mann-Whitney U test (ns, non-significant). (C-D) Spearman Rank correlations between RBD binding affinity and cell surface staining or virus capture assay at different temperatures (C), and between cell surface staining with virus capture assay at different temperatures (D). Panel A, C and D refers to data shown in Figure 3.

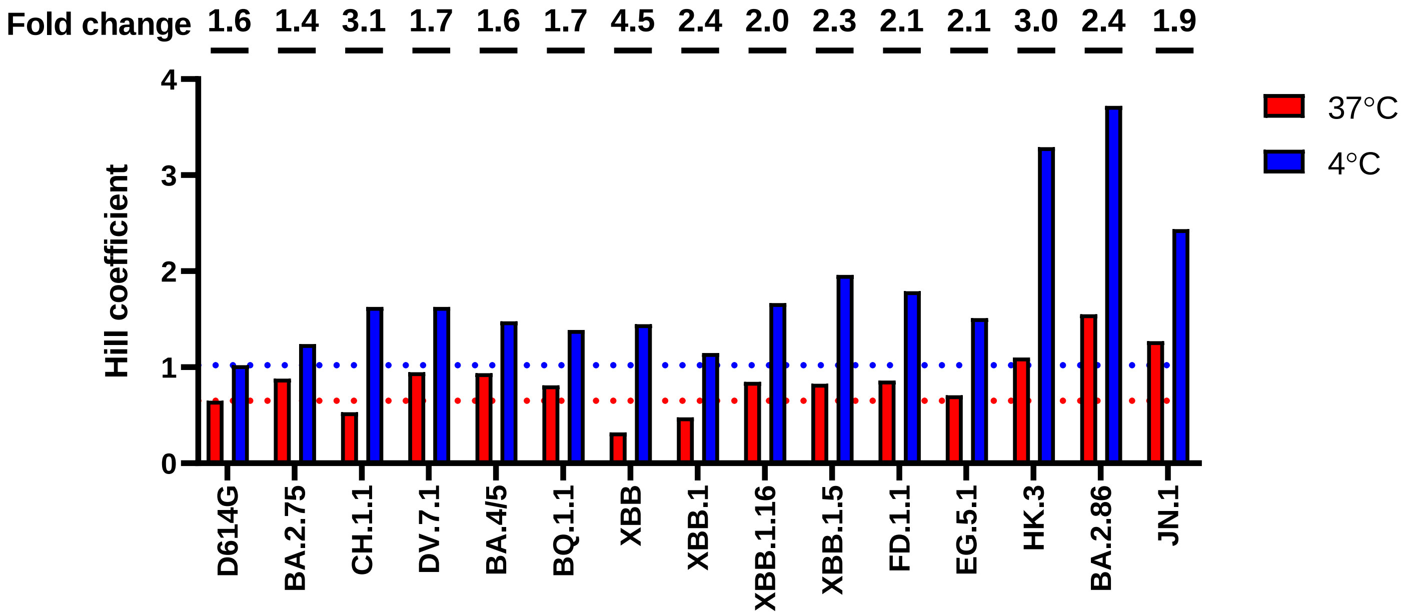

Figure S2

Low temperatures accentuate Omicron subvariant Spikes cooperativity compared to D614G
Binding of sACE2 to the Spike of D614G and several Omicron subvariants expressed at the surface of HEK293T cells was measured by flow cytometry. Increasing concentrations of sACE2 were incubated with Spike-expressing cells at 37°C (red) or 4°C (blue). Means ± SEM derived from at least three independent experiments are shown. Hill coefficients were calculated using the GraphPad software. Fold changes of Hill coefficient at 4°C vs 37°C for each Spike is shown in black.
