# Supplementary material for: Temperature-dependent Spike-ACE2 interaction of Omicron subvariants is associated with viral transmission
Source: mBio. 2024 Jul 2;15(8):e00907-24. doi: 10.1128/mbio.00907-24 (PMC11323525; doi:10.1128/mbio.00907-24)
Supplement: Supplemental Text — Supplemental methods. [file mbio.00907-24-s0002.docx]

**SUPPLEMENTAL METHODS**

**Protein expression and purification**

FreeStyle 293F cells (Invitrogen, Waltham, MA, USA) were grown in FreeStyle 293F medium (Invitrogen) and transfected with a plasmid coding for soluble ACE2 (sACE2, 1-615), ACE2-Fc (1-615) or the CV3-25 mAbs, using ExpiFectamine 293 transfection reagent, as directed by the manufacturer (Invitrogen). One week later, the cells were pelleted and discarded. Supernatants were filtered using a 0.22 µm filter (Thermo Fisher Scientific). The recombinant sACE2 protein was purified by nickel affinity columns, as directed by the manufacturer (Invitrogen). ACE2-Fc and CV3-25 mAb were purified using protein A affinity column (Cytiva, Marlborough, MA, USA), as directed by the manufacturer. Protein preparations were dialyzed against phosphate-buffered saline (PBS) and stored at -80°C in aliquots until further use. To assess purity, recombinant proteins were loaded on SDS-PAGE gels and stained with Coomassie Blue.

**Flow cytometry analysis of cell-surface staining**

Using the standard calcium phosphate method, 10 µg of Spike expressors and 2.5 µg of a green fluorescent protein (GFP) expressor (pIRES2-GFP, Clontech) were transfected into 3 × 10^6^ 293T cells. At 48h post transfection, 293T cells were stained with anti-Spike monoclonal antibodies CV3-25 (5 µg/mL) or using ACE2-Fc (10 µg/mL) for 45 min at 37℃, 25℃ or 4℃. Alternatively, to determine the Hill coefficients, cells were preincubated with increasing concentrations of sACE2 (0 to 166 nM) at 37°C or 4°C. sACE2 binding was detected using a polyclonal goat anti-ACE2 (RND systems, Minneapolis, MN, USA). AlexaFluor-647-conjugated goat anti-human IgG (H+L) Ab (Invitrogen) and AlexaFluor-647-conjugated donkey anti-goat IgG (H+L) Ab (Invitrogen) were used as secondary antibodies to stain cells for 30 min at room temperature. The percentage of transfected cells (GFP+ cells) was determined by gating the living cell population based on viability dye staining (Aqua Vivid, Invitrogen). Samples were acquired on an LSRII cytometer (BD Biosciences, Mississauga, ON, Canada) and data analysis was performed using FlowJo v10.3 (Tree Star, Ashland, OR, USA). Hill coefficient analyses were done using GraphPad Prism version 8.4.2 (GraphPad, San Diego, CA, USA). Values obtained for ACE2-Fc were normalized to CV3-25 signal and represented as a fold over D614G at 37℃.

**Virus neutralization assay**

For this, HEK293T cells were transfected with the lentiviral vector pNL4.3 R^-^E^−^ Luc and a plasmid encoding the different S glycoproteins at a ratio of 10:1 to produce SARS-CoV-2 pseudoviruses. Two days post-transfection, cell supernatants were harvested and stored at −80 °C until use. For the neutralization assays, 293T-ACE2 target cells were seeded at a density of 1 × 10^4^ cells/well in 96-well luminometer-compatible tissue culture plates (PerkinElmer, Waltham, MA, USA) 24 h before infection. Pseudoviral particles were incubated with several plasma dilutions (1/50; 1/250; 1/1250; 1/6250; 1/31250), or CV3-25 mAb (from 10µg/mL to 0µg/mL), or ACE2-Fc (from 140µg/mL to 0µg/mL) for 1 h at 37°C and were then added to the target cells followed by incubation for 48 h at 37 °C. The cells were lysed, and luciferase activity was measured as described above. The neutralization half-maximal inhibitory dilution (ID_50_) represents the plasma dilution or the protein concentration (IC_50_) to inhibit 50% of the infection of HEK293T-ACE2 cells by pseudoviruses.

**Biolayer interferometry**

Binding kinetics were performed using an Octet RED96e system (ForteBio, Fremont, CA, USA) at different temperatures (35°C, 25°C, 15°C) shaking at 1,000 RPM. Amine Reactive Second Generation (AR2G) biosensors (Sartorius, Göttingen, Germany) were hydrated in water, then activated for 300 s with a solution of 5 mM sulfo-NHS and 10 mM EDC (Sartorius) prior to amine coupling. Either SARS-CoV-2 RBDWT produced in-house or SARS-CoV-2 RBD from several Omicron subvariants (purchased from SinoBiological) were loaded into AR2G biosensor at 12.5 µg/mL at 25°C in 10 mM acetate solution pH 5 for 600 s then quenched into 1 M ethanolamine solution pH 8.5 (Sartorius) for 300 s. Loaded biosensor were placed in 10X kinetics buffer (Sartorius) for 120 s for baseline equilibration. Association of sACE2 (in 10X kinetics buffer) to the different RBD proteins was carried out for 180 s at various concentrations in a two-fold dilution series from 500 nM to 31.25 nM prior to dissociation for 300 s. The data were baseline subtracted prior to fitting, which was performed using a 1:1 binding model and the ForteBio data analysis software. Calculation of on rates (kon), off rates (koff), and affinity constants (KD) was computed using a global fit applied to all data.
